# Improving a photosynthetic bioprocess with a ubiquitous additive: Using clay powder in the cultivation of *Rhodopseudomonas palustris*

**DOI:** 10.1016/j.btre.2025.e00930

**Published:** 2025-10-08

**Authors:** Sheida Stephens, Mona Abo-Hashesh, Radhakrishnan Mahadevan, D. Grant Allen

**Affiliations:** a Department of Chemical Engineering and Applied Chemistry, University of Toronto, 200 College St., Toronto, Ontario M5S 3E5, Canada; b Faculty of Science, University of Port Said, Port Said, 42526, Egypt; c Institute of Biomaterials and Biomedical Engineering, University of Toronto, Toronto, Ontario, Canada

**Keywords:** Bioprocess, Rhodopseudomonas palustris, Purple bacteria, Photobioreactor, Biofilms

## Abstract

A key challenge in process optimization is reactor performance, particularly in light-dependent bioprocesses. While novel photobioreactor designs exist, adapting established bioreactors with simple media supplementation may provide a more practical and modular alternative to complex mechanical modifications. In this study, we evaluated the effect of supplementing 0.2 % (w/v) clay powder on acetate production by *Rhodopseudomonas palustris* grown on butyrate. Among the clay types tested, kaolin showed the most pronounced benefits, with acetate accumulation increasing by 45 % relative to controls. These improvements are attributed to a combination of enhanced light distribution and increased cellular aggregation. Photosynthetic photon flux density measurements confirmed that kaolin reduced the extent of cellular shading by increasing light scattering within the culture. Taken together, these findings demonstrate that supplementation with a widely available clay can improve light penetration in suspended phototrophic cultures, offering a novel andaccessible strategy for optimizing photosynthetic bioprocesses.

## Highlights

• Kaolin clay supplementation enhances lateral light distribution in photosynthetic cultures by increasing light scattering, as demonstrated by PPFD measurements taken perpendicular to incident light.
• The addition of silica, bentonite, and kaolin increases product concentration by 13 %, 34 %, and 45 % respectively under process conditions.
• The presence of kaolin in culture increases cellular aggregation.
• Rhodopseudomonas palustris preferentially consumes acetate over butyrate below 1 g/L.

## Graphical abstract

**Figure:**
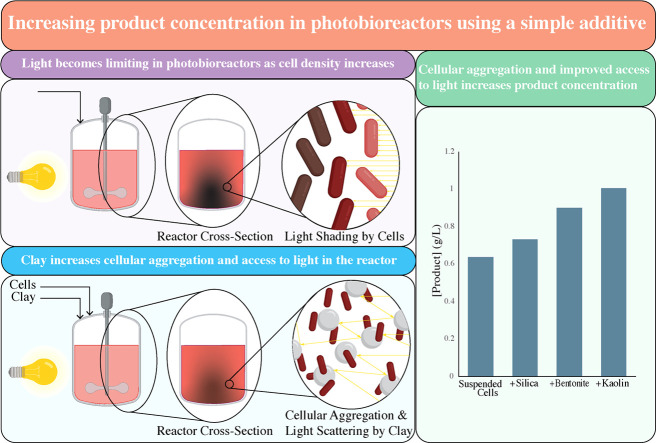

## 1 Introduction

A growing interest in sustainable bioprocesses is driven by the need to convert waste streams into valuable products [1]. Photosynthetic organisms play a key role in this effort, offering the potential to produce biofuels and other high-value compounds using light as an energy source and waste-derived feedstocks as inputs. Among these organisms, *Rhodopseudomonas palustris*, a photosynthetic purple non-sulfur bacterium, stands out due to its metabolic flexibility and ability to utilize a wide range of organic substrates, making it ideal for waste valorization.

Purple bacteria, among the oldest organisms on earth, are native hydrogen producers, and perform anoxygenic photosynthesis using a more ancient form of photosynthesis than the more common oxygenic type performed by plants and algae [2,3]. They thrive in many types of wastewater streams, as their preferred mode of growth is photoheterotrophy [4], which uses organic carbon sources as a feedstock, and have been used in the cleanup of streams for olive oil mills [5,6], swine refuse wastewater [7], crude glycerol [8], and bioremediation of aromatic compounds [9]. They can thus produce the valuable clean fuel of hydrogen from waste. They have also, in recent years, been identified as an unconventional tool for bio-engineering as they produce an abundance of electrons from this specialized photoheterotrophy that can then be diverted to non-native products via metabolic engineering [1]. *R. palustris* has been used as a chassis for the production of numerous non-native products with marketable value including butanol [10], β-carotene [11], and squalene [12].

Given the interest in using *R. palustris* as a chassis for biotechnology applications, when designing systems for light-dependent bioprocesses such as these, light delivery becomes a primary concern. Conventional bioreactors are typically suboptimal for cultivating photosynthetic cells, as lighting is exterior and the center of the reactor receives less light than the walls due to cellular shading and light attenuation. Even with mixing, light becomes limiting with higher cell densities in these systems. This limitation reduces the viability of established bioreactor designs in bioprocess development, often necessitating the use of novel, potentially untested designs. For this reason, researchers have been working on developing specialized methods for improving light delivery in closed systems for photosynthetic organisms mainly focusing on minimizing depth of the reactor from the light source [[13], [14], [15]]. One such means of accomplishing this involves the utilization of cellular immobilization such as the formation of a biofilm on light waveguides: organisms can grow directly on the surface of a light source allowing for direct access to light and reduced light shading inside of a reactor compared to suspended cell processes [16]. However, even when the surface is not a light source, there are benefits to aggregated cultivation.

One benefit observed in processes that used cellular immobilization is longer production phases. For example, in yeast immobilized in a 3D printed polymer, cells produced product for over a year [17]. With *R. capsulatus*, another type of purple bacteria, when immobilized in agar, cells produced hydrogen for over 70 days [18]. With *R. palustris*, studies have shown that with the introduction of a surface for cell adsorption, an increase in hydrogen yield was observed in the same reactor for the equivalent amount of feedstock. Some surfaces studied included clay, agar beads, and activated carbon [19,20]. While it has been proposed that increased product yield in immobilized systems is due to higher cellular density compared to suspended cultures [21] and lower metabolic demand in immobilized cells [17], these findings warrant further investigation to better understand and exploit the underlying mechanisms.

In this study, we impeded native *R. palustris* hydrogen production to test a theory: if an increase in hydrogen is observed with the introduction of clay, then an increase in other natively produced products, such as organic acids, should also be observed when compared to the condition with no clay additives. The primary objective of this study is thus to investigate the effect of clay particle addition on the growth and physiology of *R. palustris*, with the specific goal of evaluating clay as an additive to enhance bioreactor efficiency. In this study, we will be investigating the influence of clay on *R. palustris* in batch-case bioreactors specifically while consuming butyrate.

## 2 Methods

### 2.1 Metabolism

**Fig. 1:**
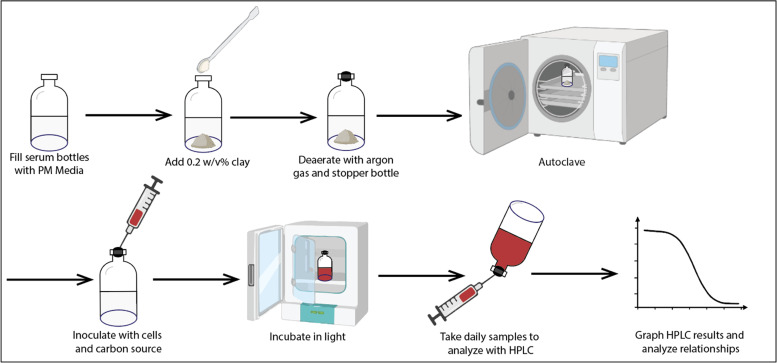
Experimental procedure: fill glass serum bottles with 50 ml PM, add 100 mg of clay, deaerate bottles with argon gas, stopper bottles, and autoclave. Four conditions were set up: cultures supplemented with silica, bentonite, kaolin, or no supplementation. After inoculating with *R. palustris* cells at an OD660 of 0.1, incubate bottles, and take daily samples for OD measurements and for HPLC analysis. All experiments were conducted with a minimum of triplicate replicates (figure created using BioRender).

While consuming butyrate photoheterotrophically under hydrogen-inhibiting conditions, *R. palustris* breaks down butyrate in its central metabolism and produces CO_2_ and acetate as its products [22]. The produced CO_2_ is then taken back up by the cells to be used as electron sinks via the Calvin cycle. Butyrate is less reduced than the cell biomass so *R. palustris* needs to be supplemented with additional electron sinks— not enough can be produced by the cells to avoid a lethal redox imbalance, which is not the case when it consumes other carbon sources that are more reduced than the cellular biomass such as succinate or fumarate [22]. However, without the supplementation of additional CO_2_ or sodium bicarbonate and without conditions that allow for hydrogen production, cells will not grow when provided with butyrate as their carbon source because the cell runs out of electron sinks for the abundance of electrons available from its central metabolism and photosynthesis [22]. Therefore, in our study, we supplemented media with known quantities of butyrate as the carbon source, sodium bicarbonate as an additional electron sink, and ammonium nitrate to encourage photoheterotrophic growth conditions and prevent the production of hydrogen. Specifically, we characterized the cellular growth, butyrate consumption, and acetate production with and without addition of clay particles and under two different light intensities to identify the impact of these additives. Our overall methodology is shown in Fig. 1.

### 2.2 Clays

Ceramsite clay balls (Wohohoho Grow Media, 4 mm, Amazon) were selected due to ease of public accessibility and simplicity of separation from the bulk fluid. Silica gel (CAS-No 112,926–00–8, 717,185, Sigma-Aldrich), kaolin (CAS-No 1332–58–7, K7375, Sigma-Aldrich), and bentonite (CAS-No 1302–78–9, 285,234, Sigma-Aldrich) clays were selected to supplement *R. palustris* cultures. Each of these are used in a variety of different applications and were selected due to their ubiquitousness in both industry and nature [[23], [24], [25]].

### 2.3 Strain and culture conditions

*R. palustris* strain CGA009 was obtained from ATCC (ATCC BAA-98, *Rhodopseudomonas palustris* (Molisch) van Niel). The medium used was Photosynthetic Medium (PM) based on the directions of Kim and Harwood [26]. Concentrated stock solutions of 100 g/l sodium butyrate, 2 M sodium acetate, and 160 g/L sodium bicarbonate were filter-sterilized and supplemented to PM after autoclaving.

Precultures were grown from plates at 30 °C in 50 mL culture tubes (Falcon 352,070, Fisher Scientific) filled to the top with PM supplemented with 1 g/L sodium butyrate and 1.6 g/L sodium bicarbonate or 20 mM sodium acetate with no bicarbonate addition and grown statically to encourage anaerobicity in light until bacteria reached an OD at 660 nm (OD660) of 0.6–1.0.

Under experimental conditions (Fig. 1), 50 mL PM was added to standard 125 mL clear glass serum bottles with a rubber stopper and aluminum crimp. In each bottle containing clay, 100 mg of clay was measured and added into the PM. For the clay ball condition, approximately 70 clay balls were counted out totalling 14 g dry weight.

Anoxygenic conditions were subsequently achieved by deaerating each bottle with argon gas and autoclaving. Each bottle was then inoculated with the equivalent of 0.1 OD660 *R. palustris* and placed equidistantly from a natural white light LED source (IT5–40K12–120 V, Super Bright LEDs Inc.) in a large floor incubator (Innova 4300, New Brunswick Scientific) at 30 °C.

To encourage flocculation of cells and adsorption to supplemented surfaces, no carbon source was supplemented for the first 24 h (adapting the method outlined in ASTM International [27]) after which the equivalent of 2.6 g/L sodium butyrate and 1.6 g/L sodium bicarbonate or 1.8 g/L sodium acetate was added to each bottle.

Light intensity to each bottle was measured using a portable spectrometer (LI-180 Spectrometer, LI-COR Environmental). Specifically, the photosynthetic photon flux density (PPFD) in the range of 400–700 nm was measured for this. Each experimental run lasted 10 days. Samples from each bottle were taken daily. From those samples, OD660 was measured on a spectrophotometer (AquaMate 7100-Vis Spectrophotometer, Thermo Scientific) and samples were filtered and prepared for HPLC analysis using syringe filters (0.22 μm, Fisherbrand).

### 2.4 Quantification of organic acids using HPLC

HPLC analysis was used for the measurement of organic acids within the process media. This was done in an UltiMate 3000 system (ThermoFisher Scientific) using an Aminex HPX-87H column (Bio-Rad Laboratories). Each run was at 50 °C using 10 mM sulfuric acid as the carrier fluid flowing at 0.6 mL/min with 35 min between injections. The HPLC is equipped with both a refractive index detector and ultraviolet detector (run at 214 nm).

### 2.5 Microscopy

In order to observe cellular and clay characteristics after cultures had fully matured, end point samples were taken from each bottle following 10 days of growth and light field microscopy was performed on each using the brightfield setting of an Olympus BX51 microscope at 100x magnification. Photos of observations were recorded in Olympus cellSens Dimension software.

### 2.6 Quantification of aggregates

Fiji [28] was used to quantify aggregates observed under the microscope. For each experimental condition, 6–9 images were collected from endpoint samples. The analysis procedure involved setting the scale, converting images to 8-bit grayscale, manually thresholding, and analyzing all identified aggregates [29]. Aggregates for our purposes were defined as two or more connected cells; single cells were excluded by setting the minimum aggregate size to 0.1 µm^2^. Quantified aggregates are outlined in red overlaid on the raw grayscale microscope images, as shown in Supplemental Figs. S2-S5.

### 2.7 Test of light distribution

To assess the light-reflective properties of kaolin clay, an experiment was conducted to measure light distribution under different conditions (Supplementary Fig. S6). Four treatments were prepared in serum bottles: (i) PM medium alone, (ii) PM medium with *R. palustris* cells, (iii) PM medium with *R. palustris* cells and kaolin clay, and (iv) PM medium with kaolin clay alone. Each serum bottle contained 50 mL of PM medium. For conditions (ii) and (iii), *R. palustris* cells were added to achieve a final optical density of OD660 = 1.0. For conditions (iii) and (iv), 100 mg of kaolin clay was added.

Each serum bottle was placed directly on top of a flat LED flashlight (Apple iPhone 8 Plus), and photosynthetically active photon flux density (PPFD, in µmol·m⁻²·s⁻¹) was measured from the side of each bottle using a portable spectrometer (LI-180 Spectrometer, LI-COR Environmental).

### 2.8 Statistical analyses

The Area Under the Curve (AUC) method [30,31] was used to assess statistical differences in the time-course profiles of acetate and butyrate concentrations for each clay condition relative to the suspended control. Instantaneous concentration values were first calculated, and AUC was determined using the trapezoidal integration method, representing the total amount of substrate accumulated over the course of the experiment. This approach was chosen over endpoint yield or concentration as it reduces the impact of short-term fluctuations and captures the cumulative effect, which is particularly relevant in the case where product is subsequently consumed. A one-way ANOVA was performed separately for acetate and butyrate to test for significant differences among group means, with clay condition as the independent variable and AUC as the dependent variable. Where significance was detected, a post hoc Games-Howell test—robust to unequal variances—was used to identify which conditions differed significantly from the suspended control.

To compare aggregate area distributions from Fiji analyses, the Mann-Whitney U test [32] was utilized. This non-parametric test was chosen over the *t*-test to avoid assumptions of normality.

To compare PPFD measurements, a one-way ANOVA was performed followed by a post-hoc Games-Howell test to identify which pairs of means differed significantly from the other.

## 3 Results and discussion

### 3.1 Preliminary trials using hydroponic materials

**Fig. 2:**
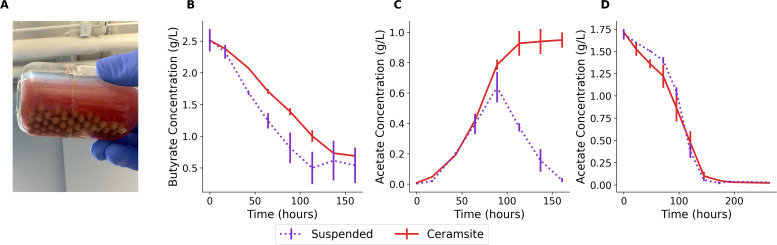
Time-course profiles of preliminary experiments using ceramsite clay balls relative to suspended controls. (A) Ceramsite clay balls in culture medium with *R. palustris*. (B) Butyrate consumption over time. (C) Acetate production during butyrate oxidation. (D) Acetate consumption over time. Solid red lines represent ceramsite-immobilized cultures; dotted purple lines represent suspended cell controls. Data represent means ± standard error (*n* = 3).

Inspired by the open science movement, which seeks to make research more accessible through readily available materials and open-source laboratory equipment [[33], [34], [35]], we conducted preliminary experiments of clay supplementation using clay balls that can be readily purchased from commercial vendors (Fig. 2a). These clay balls are typically marketed for hydroponics or for maintaining soil moisture in plant systems, but here they were repurposed for application in this phototrophic bioprocess. Beyond their accessibility, another practical advantage of clay balls is the simplicity of their separation and reuse.

When cultures were supplemented with butyrate, the first notable observation was that less substrate was consumed in clay-supplemented cultures compared to unsupplemented suspended controls (Fig. 2b). A second observation was that acetate, the product of *R. palustris* when consuming butyrate, was subsequently depleted in suspended cultures but continued to accumulate in clay-supplemented cultures, reaching higher maximum concentrations than in the controls (Fig. 2c). This trend was consistent with prior reports of enhanced hydrogen production, where higher hydrogen yields were obtained from the same substrate load when clay was supplemented in culture [36].

To confirm that these effects were not due to clay directly inhibiting acetate uptake, acetate was provided as the sole carbon source without butyrate supplementation (Fig. 2d). Under these conditions, *R. palustris* fully depleted acetate, with no significant differences observed between suspended and clay-supplemented cultures.

Taken together, these preliminary findings motivated the design of a more rigorous experimental scheme using fully characterized clay powders to better elucidate the mechanisms underlying the observed effects.

### 3.2 With 37 % lower light intensity, cells grew 2.7 times slower

Given our objective of analyzing how the addition of clay particles affects the growth and physiology of *R. palustris*, we designed the study to be conducted at two different light intensities. This approach allowed us to isolate the effects of light on growth, ensuring they could be distinguished from those caused by the addition of clay particles. We anticipated a significant difference between cultures with and without clay, but we also observed light-dependent and organism-specific differences. Notably, we observed variations in growth rate, carbon source preference, carbon source utilization, clay-dependent aggregation, and enhanced product formation. These findings are discussed in the following sections.

**Fig. 3:**
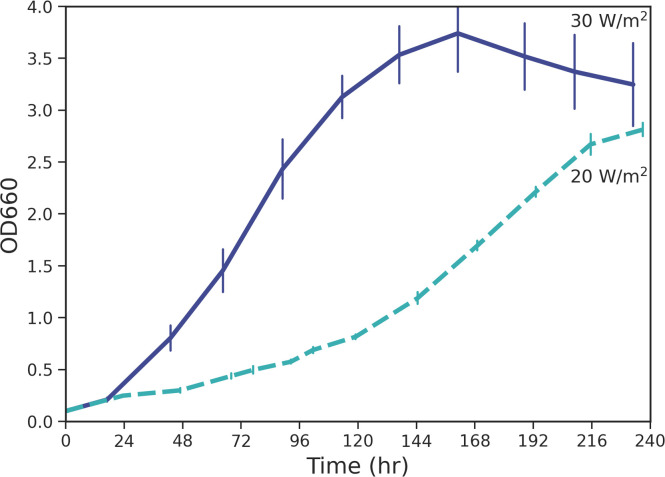
Growth of suspended *R. palustris* cells over time when irradiated with white light at two different intensities: 30 W/m^2^ and 20 W/m^2^. At 30 W/m^2^, *R. palustris* has a doubling time of 14 h. At 20 W/m^2^, *R. palustris* has a doubling time of 38 h. Error bars shown are the standard error of triplicate and quadruplicate samples respectively.

The cells were grown at two different growth rates, which was accomplished by manipulating the lighting array of the incubator to achieve different average light intensities as measured by a portable spectrometer. Although the light intensity utilized in this study is not near the peak of 200 W/m^2^ for *R. palustris* [37], we referred to 30 W/m^2^ as “high” light intensity (LI) and 20 W/m^2^ as “low” LI for simplified reference to these conditions. As shown in Fig. 3, at 30 W/m^2^, *R. palustris* grew faster with a calculated doubling time of 14 h than it did at 20 W/m^2^ with a calculated doubling time of 38 h. Large increases in growth rate are expected when light intensity is below peak intensity [37], which is the case in this study.

In addition to the reduced absorption of photons by cells at lower light intensities, light intensity also influences gene expression in *R. palustris*; under low light conditions, cells increase the production of light-harvesting complexes and pigments, requiring greater metabolic effort and reducing growth rate [38]. In this study, light intensity was an essential process parameter to ensure that observed differences between clay and suspended cultures were not only attributed to variations in light intensity—evident through cellular growth rates—since light profoundly affects cellular behavior at the genetic level.

### 3.3 R. palustris *preferentially consumed acetate over butyrate past a concentration threshold*

Both acetate and butyrate are carbon sources for *R. palustris,* but the cells consume them each differently. While the consumption of both carbon sources results in the release of CO_2_ and its reuptake via the Calvin cycle, the consumption of butyrate relies more heavily on the Calvin cycle than acetate does, which relies more heavily on the cell’s central metabolism [22]. The total flux of electrons through the cell that reduce electron carriers is 1.5 times higher when consuming butyrate than acetate [22]. This explains why bicarbonate is required in the medium as an additional electron sink during the consumption of butyrate, but it also highlights butyrate as a more metabolically challenging carbon source for the cells to utilize.

**Fig. 4:**
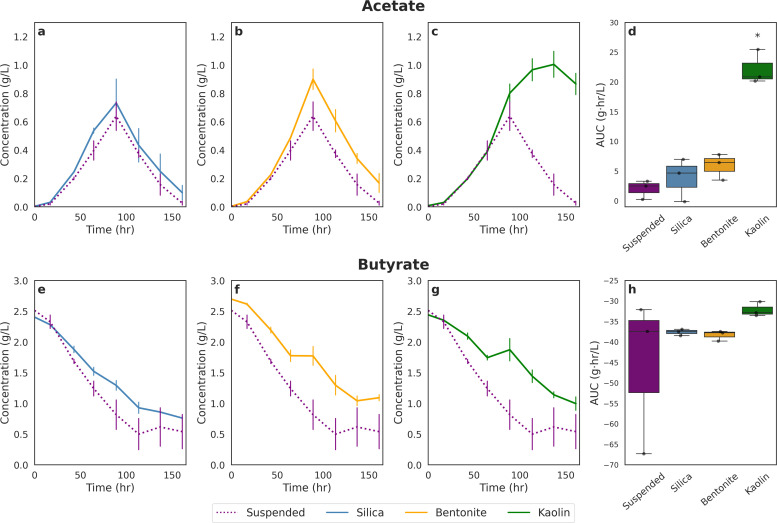
Time-course profiles and integrated metabolite production for each experimental condition irradiated at 30 W/m^2^. Panels a-c show the time-course of acetate concentrations and Panels e-g show the time-course of butyrate concentrations under silica, bentonite, and kaolin treatments, respectively, compared to the suspended control (dotted line). Values represent means ± standard error (*n* = 3). Panels d & h display the area under the curve (AUC) for each condition, with data presented as boxplots overlaid with individual replicates. Statistical significance was assessed using one-way ANOVA followed by Games-Howell post-hoc test; asterisks (*) indicate conditions significantly different from the Suspended control (*p* < 0.05).

**Fig. 5:**
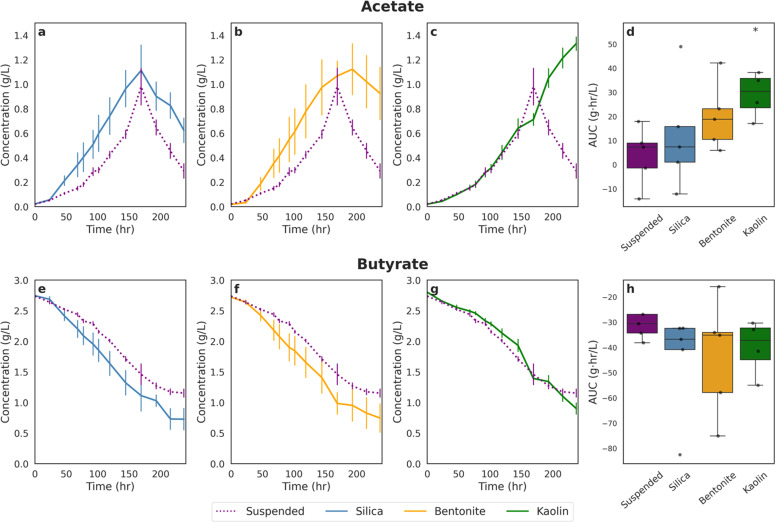
Time-course profiles and integrated metabolite production for each experimental condition irradiated at 20 W/m^2^. Panels a-c show the time-course of acetate concentrations and Panels e-g show the time-course of butyrate concentrations under silica, bentonite, and kaolin treatments, respectively, compared to the suspended control (dotted line). Values represent means ± standard error (*n* = 4). Panels d & h display the area under the curve (AUC) for each condition, with data presented as boxplots overlaid with individual replicates. Statistical significance was assessed using one-way ANOVA followed by Games-Howell post-hoc test; asterisks (*) indicate conditions significantly different from the Suspended control (*p* < 0.05).

Figs. 4 and 5 illustrate how butyrate is consumed and how acetate is produced by *R. palustris* supplemented with clay compared to suspended cultures (no clay supplementation) over time at the two different light intensities. Looking at the suspended cell case for both High and Low LI, butyrate consumption decreased linearly until approximately 1 g/L butyrate concentration when it began to level off. At the same point where the butyrate concentration levelled off, acetate concentration began to decline indicating a switch in carbon source. The same trend was observed for cultures supplemented with silica and bentonite clays at both High and Low LI. This may have either been caused by the dwindling abundance of substrate like butyrate or bicarbonate or by the presence of increased concentrations of a more easily consumable carbon source. However, there is more evidence for the latter: when *R. palustris* is fed 1 g/L butyrate to start with (Supplementary Fig. S1), minimal acetate generation is visible by HPLC analysis, which indicates that acetate is being consumed as it is being produced and that it is a preferred carbon source for the strain.

The only exception to this trend was observed under the kaolin-supplemented condition. In this case, acetate concentrations remained elevated while butyrate continued to decrease, indicating that the expected carbon switch did not occur. The same pattern was observed under both the High LI and Low LI conditions. A similar outcome was also noted in the preliminary study with clay ball supplementation.

### 3.4 Increased consumption of butyrate with lower light intensity

Similar to our preliminary findings with clay balls, under High LI conditions (Fig. 4), supplementation with clay minerals resulted in reduced butyrate consumption relative to the suspended control, despite concurrently higher acetate production—particularly in the kaolin-supplemented condition. Across all clay-supplemented cultures, residual butyrate concentrations remained elevated throughout the experimental period. Specifically, cultures supplemented with silica (Fig. 4e), bentonite (Fig. 4f), and kaolin (Fig. 4g) exhibited overall higher butyrate concentration throughout the experimental runs. AUC analysis, however, did not find these differences significant (Fig. 4h).

In contrast, under Low LI conditions (Fig. 5), a different trend emerged. Cultures supplemented with silica (Fig. 5e) and bentonite (Fig. 5f) consumed more butyrate than the suspended control, while the kaolin-supplemented condition (Fig. 5g) showed a similar butyrate consumption profile to the suspended culture. However, statistical analysis using the AUC method revealed no significant differences in butyrate consumption between any of the clay-supplemented conditions and the suspended control (Fig. 5h). While not found to be significantly different, this observation is noteworthy given the observed differences in the growth trends under the same conditions.

The most apparent difference between the High LI and Low LI experimental runs is the light intensity itself, which not only influences the availability of energy for phototrophic growth but also affects the growth rate and metabolic behavior of *R. palustris*. As previously discussed, *R. palustris* responds to reduced light levels by upregulating the synthesis of light-harvesting complexes and photosynthetic pigments [38], which supports continued energy acquisition under suboptimal lighting but also results in slower growth rate. Slower growth is typically associated with an increased allocation of resources toward cellular maintenance and stress adaptation, rather than biomass accumulation. As a result, cells may exhibit elevated metabolic demands for energy and reducing equivalents, requiring a greater uptake of substrates such as butyrate to sustain basal physiological functions.

### 3.5 Clay supplementation encouraged cellular flocculation

Most microbial cells in the natural environment grow within biofilms [39], many of which are on clay surfaces. In *R. palustris*, it has been shown that cells use aggregation, a form of biofilm, as a tool when growing photoheterotrophically and even more so under difficult growth conditions such as nitrogen-fixation conditions or high osmotic stress [40]. This aggregation is not observed when it grows chemoheterotrophically or photoautotrophically [40], which are growth conditions that are not generally as redox sensitive as photoheterotrophy. This is a strong indication that there is an evolutionary advantage to cellular aggregation in *R. palustris* not only under stressful growth conditions but also when it grows photoheterotrophically. It is probable that there are bacterial cell-cell interactions during aggregation, which become important during photoheterotrophy.

In aggregates, cells of even single-strain communities begin to act as multi-cellular organisms such as by the division of labour and sharing energy resources [41]. Considering the amount of energy available to *R. palustris* during photoheterotrophy and the delicate balance of redox reactions, these types of benefits would provide an advantage to the cells. The addition of clay encourages this aggregation due to charged surfaces for cellular adsorption [[42], [43], [44], [45]].

**Fig. 6:**
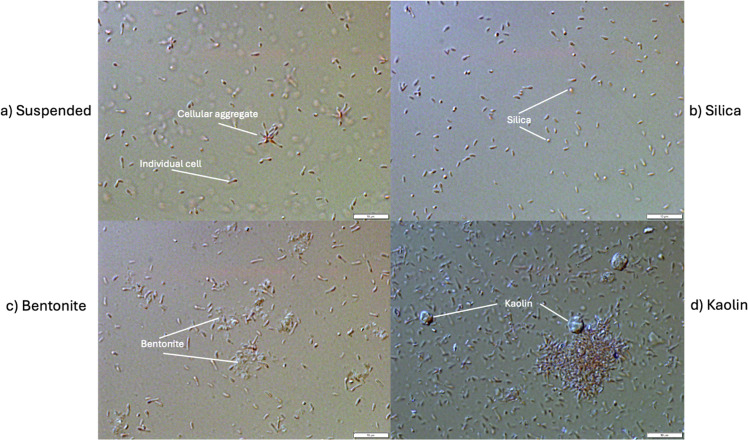
Brightfield microscopy images of *R. palustris* cultures at the experimental endpoint. All images were captured at 100× magnification with a scale bar of 10 µm. (a) Suspended culture without clay supplementation, showing only *R. palustris* cells, both as individual rods and small aggregates. (b) Silica-supplemented culture, with spherical silica particles visible; minimal cellular aggregation is observed. (c) Bentonite-supplemented culture, where bentonite appears as diffuse, cloud-like material surrounded by *R. palustris* cells. (d) Kaolin-supplemented culture, with kaolin particles visible as small, rock-like structures surrounded by densely aggregated *R. palustris* cells.

Representative brightfield microscopy images of *R. palustris* are presented in Fig. 6. The rod-shaped morphology of *R. palustris* cells is clearly visible, along with particulate matter corresponding to the added silica, bentonite, and kaolin clay supplements. In the bentonite- (Fig. 6c) and kaolin-supplemented cultures (Fig. 6d), cells appear to preferentially localize around the clay particles, suggesting potential interactions or surface-associated clustering. Visible aggregates are present in the suspended control (Fig. 6a), as well as in the bentonite and kaolin conditions, with the latter displaying particularly pronounced aggregation. However, the silica-supplemented cultures (Fig. 6b) exhibit minimal evidence of cellular clustering, with most cells appearing individually dispersed.

**Fig. 7:**
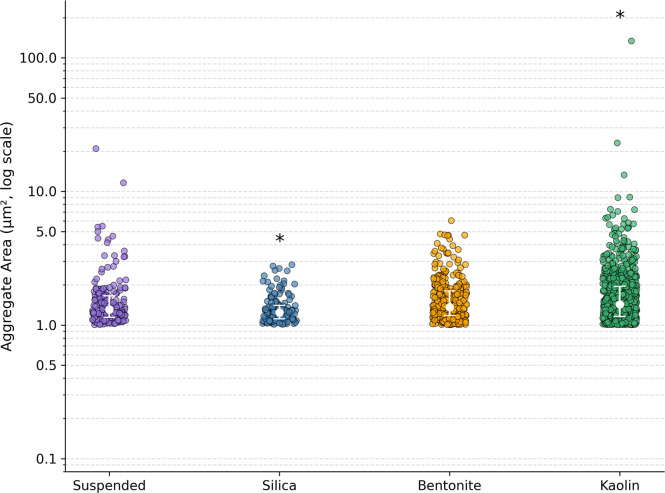
Aggregate area distributions under four experimental conditions: suspended, silica, bentonite, and kaolin. Each point represents the area of a single aggregate, quantified via image analysis of microscopy images using Fiji. Statistical comparisons were performed against the suspended condition using the Mann–Whitney U test; significant differences (p < 0.05) are indicated by asterisks above the respective groups. Non-significant comparisons are not labeled.

To assess whether aggregation differed significantly among conditions, all qualitative microscopy images were processed and converted into quantitative data using Fiji. The resulting analyses are presented in Fig. 7. As anticipated, the kaolin-supplemented condition exhibited a significantly higher degree of aggregation compared to the suspended control. In contrast, the silica-supplemented condition also showed a statistically significant difference from the suspended condition; however, this difference was attributed to a markedly lower frequency of aggregates. No statistically significant difference in aggregation was observed between the suspended and bentonite-supplemented cultures. However, it is important to note that clay surfaces were excluded from the Fiji-based image analysis, which focused specifically on multicellular aggregation rather than cell–clay surface interactions.

Kaolin has previously been demonstrated to be a strong adsorption surface for microbial cells [42,43]. Clays in general, but especially kaolin, allow for cation exchange with bacterial cells and the availability of surfaces for adsorption encourages bacterial interaction between cells [43,45]. This provides an advantage for cellular growth and survivability even in hostile or adverse growth environments [[45], [46]].

### 3.6 The presence of clay increased acetate concentration in culture

Under High LI conditions, cultures supplemented with clay minerals exhibited increased acetate concentration relative to the suspended control (Fig. 4). On average, silica supplementation resulted in a 14 % increase in acetate concentration compared to suspended cultures (Fig. 4a). Bentonite supplementation yielded a 34 % increase (Fig. 4b), while kaolin supplementation led to the most pronounced effect, with a 45 % increase in acetate concentration (Fig. 4c). To assess the statistical significance of these differences, the AUC method was employed (Fig. 4d). Among the three clay types, only the kaolin-supplemented condition exhibited a statistically significant increase relative to the suspended control. Although the silica- and bentonite-supplemented cultures showed higher mean acetate levels, these differences were not statistically significant.

A similar pattern was observed under Low LI conditions, wherein clay-supplemented cultures exhibited elevated acetate concentration relative to the suspended control. Specifically, silica-supplemented cultures demonstrated a 13 % increase in acetate concentration compared to suspended cultures (Fig. 5a), while bentonite-supplemented cultures showed a 14 % increase (Fig. 5b), and kaolin-supplemented cultures exhibited the most pronounced effect with a 30 % increase (Fig. 5c). Similar to the high LI condition, statistical analysis using the AUC method determined a statistically significant difference between the kaolin and suspended conditions.

The observed differences in acetate production among clay-supplemented cultures may be attributed to multiple mechanisms, acting either independently or in combination. Several reports have described clay–microbe interactions that influence metabolism by altering the cellular microenvironment. Some attribute these effects to surface-level ionic interactions between bacteria and clay, while others describe cellular immobilization on clay surfaces [47]. Additional studies have reported that microbial colonization of light-exposed ceramic surfaces typically begin with the establishment of phototrophic communities, such as cyanobacteria and microalgae [48]. Drawing on these reports and our own findings, we hypothesized two potential mechanisms: (i) cellular flocculation and aggregate formation and (ii) modifications in light distribution resulting from the presence of clay particles. Each of these hypotheses is considered as follows.

*(i) Cellular Flocculation and Aggregation.* As shown in Fig. 6, varying degrees of cellular aggregation were observed across all conditions. In the suspended control (Fig. 6a), *R. palustris* cells occasionally adhered end-to-end, forming small clusters. In silica-supplemented cultures (Fig. 6b), cells were observed to localize around silica particles, though little aggregation beyond this was evident. In contrast, bentonite-supplemented cultures (Fig. 6c) exhibited some cell clustering along bentonite surfaces, while the kaolin-supplemented cultures (Fig. 6d) displayed the most pronounced aggregation, with cells forming large multicellular clusters both in association with kaolin particles and independently.

The degree of aggregation appears to correlate qualitatively with the extent of acetate production, particularly in the case of kaolin, which exhibited both the most substantial aggregation and the highest acetate yield. However, aggregation was also observed in the suspended control, suggesting that clustering alone is unlikely to fully explain the observed enhancements. While not conclusive, it is worth noting that clay-induced aggregation has previously been reported to promote cell–cell interactions and biofilm formation in bacterial systems [47], which may be relevant to the enhanced metabolic activity observed here.

*(ii) Enhanced Light Distribution via Clay Particles.* The final and most compelling explanation involves the influence of clay particles—particularly kaolin—on the optical environment of the cultures. In botanical applications, kaolin has been used as a foliar spray to enhance light penetration into plant canopies [[49], [50], [51], [52]]. By increasing surface reflectivity, kaolin redirects sunlight toward interior leaves that would otherwise receive limited exposure, while simultaneously shielding outer leaves from excessive irradiation and photodamage. This results in an overall increase in the plant's photosynthetic capacity, despite reduced activity in the exterior leaves.

Kaolin’s light-scattering capabilities are also well-established in non-biological contexts. In the paint industry, it is commonly used to improve opacity through enhanced light dispersion [24]. Similarly, in ceramic glazes, high kaolin content contributes to more uniform coloration, a result of its capacity to scatter light effectively [24].

We propose that a similar mechanism may be operative in our photosynthetic bacterial cultures. Specifically, kaolin particles may enhance the internal distribution of light by scattering incident photons more uniformly throughout the culture medium, thereby increasing the effective photon flux available to individual cells.

**Fig. 8:**
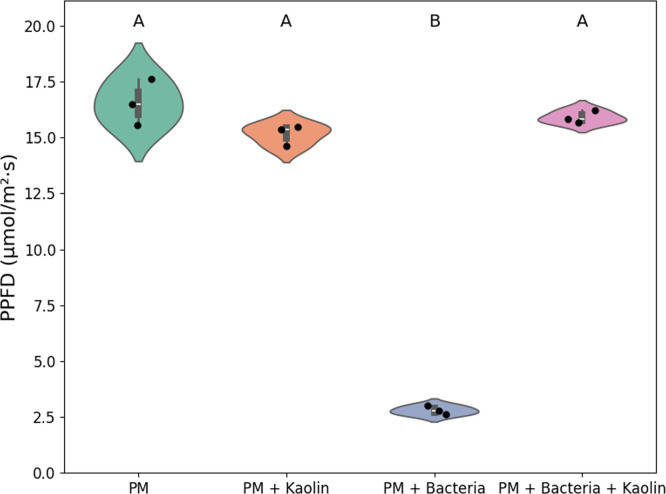
Violin plot showing photosynthetic photon flux density (PPFD) measurements across four experimental conditions: Medium (PM), Medium + Kaolin, Medium + Bacteria, and Medium + Bacteria + Kaolin. Each violin represents the distribution of replicate measurements (*n* = 3), with embedded boxplots indicating medians and interquartile ranges. Individual data points are overlaid as black dots. One-way ANOVA revealed a significant effect of condition on PPFD (*F* = 379.17, *p* < 0.0001). Post-hoc Games-Howell testing identified significant differences, summarized using compact letter display above each group. Groups sharing the same letter are not significantly different (*p* > 0.05). Notably, the PM + Bacteria condition exhibited significantly lower PPFD than all other conditions indicating light attenuation caused by bacterial presence, while kaolin supplementation restored PPFD to levels indistinguishable from controls.

To evaluate this hypothesis, an experiment was conducted in which PPFD was measured through the side of clear glass bottles when incident light was irradiated from below (Supplementary Fig. S6). As shown in Fig. 8, the addition of *R. palustris* cells to the medium resulted in significant light attenuation, consistent with shading caused by cellular biomass. However, when kaolin clay was added to a culture containing the same cell concentration, PPFD measurements were comparable to those of clear medium, indicating that light transmission was effectively preserved.

This finding supports the hypothesis that kaolin improves the optical environment within photosynthetic cultures by maintaining photon availability, even under turbid conditions. By preserving lateral light transmission, kaolin may alleviate one of the key limitations in photobioprocesses—non-uniform light distribution—thereby contributing to the observed increases in acetate production.

## 4 Conclusions

In this study, we investigated the effects of clay supplementation on *R. palustris* cultures grown on butyrate. Several key findings emerged, including some specific to *R. palustris*: acetate was preferentially consumed over butyrate beyond a concentration threshold, and butyrate consumption increased under lower light intensity. Other observations may extend to broader microbial systems, such as the enhanced cellular aggregation and increased production of metabolic products (in this case acetate) in the presence of clay. These trends were consistent across both high and low light intensity conditions, indicating that clay supplementation was the underlying driver of the observed differences. This is also consistent with what has previously been observed in hydrogen-producing *R. palustris* cultures supplemented with clay.

We propose that these effects are primarily associated with enhanced cell aggregation and improved light distribution within the culture. The most significant outcome of this study is the demonstration that kaolin improves light availability in suspended phototrophic cultures. This highlights a novel application of clay in photobioprocess engineering.

While clay supplementation of microbial cultures has previously been proposed as a means of enhancing microbial capabilities [47], our finding that kaolin can improve photobioprocesses is novel. The addition of clay particles—especially kaolin—offers a promising strategy to enhance light distribution in dense cultures. By leveraging both the light-scattering properties of clay and its promotion of cellular aggregation, this approach provides a scalable alternative to mechanical photobioreactor redesigns for improving performance in photosynthetic bioprocesses. We encourage further studies with additional phototrophic systems to establish the broader generalizability of this effect and to explore its potential for integration into phototrophic processes at scale.

## Disclosure

All the authors declared no competing interests.

## CRediT authorship contribution statement

**Sheida Stephens:** Writing – original draft, Visualization, Resources, Project administration, Methodology, Investigation, Formal analysis, Data curation, Conceptualization. **Mona Abo-Hashesh:** Resources. **Radhakrishnan Mahadevan:** Writing – review & editing, Supervision, Resources, Project administration, Conceptualization. **D. Grant Allen:** Writing – review & editing, Supervision, Resources, Project administration, Conceptualization.

## Declaration of competing interest

The authors declare that they have no known competing financial interests or personal relationships that could have appeared to influence the work reported in this paper.

## Data Availability

Data will be made available on request.

## Appendix Supplementary materials

Image, application 1

Image, application 2
