# Supplementary material for: Improving a photosynthetic bioprocess with a ubiquitous additive: Using clay powder in the cultivation of Rhodopseudomonas palustris
Source: Biotechnol Rep (Amst). 2025 Oct 8;48:e00930. doi: 10.1016/j.btre.2025.e00930 (PMC12550322; doi:10.1016/j.btre.2025.e00930)
Supplement: Supplementary file 1 [file mmc1.docx]

**Supplementary Figures**


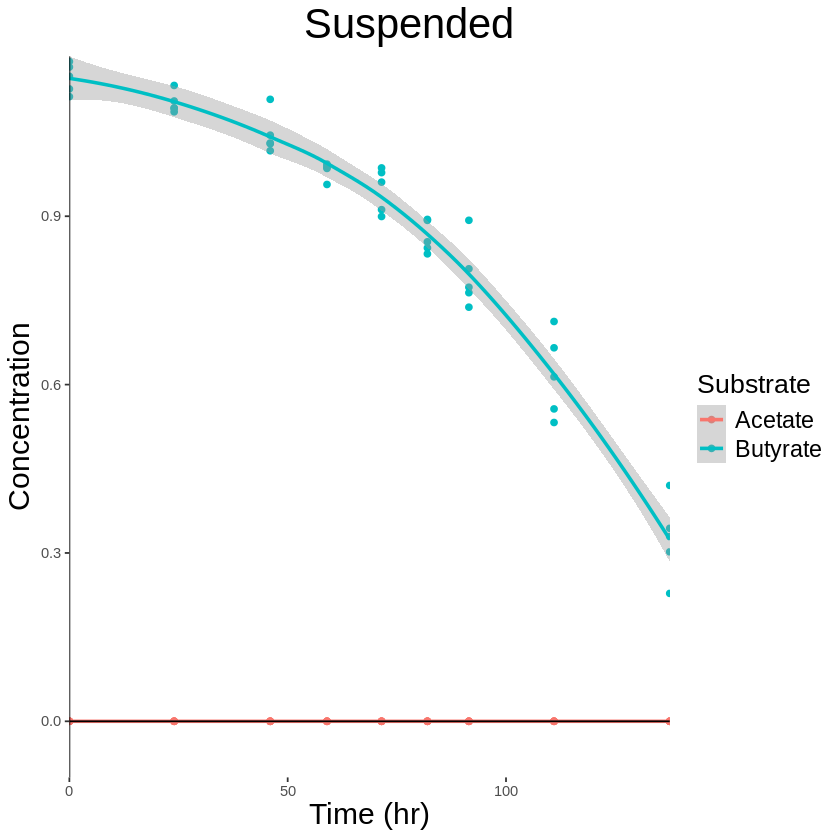

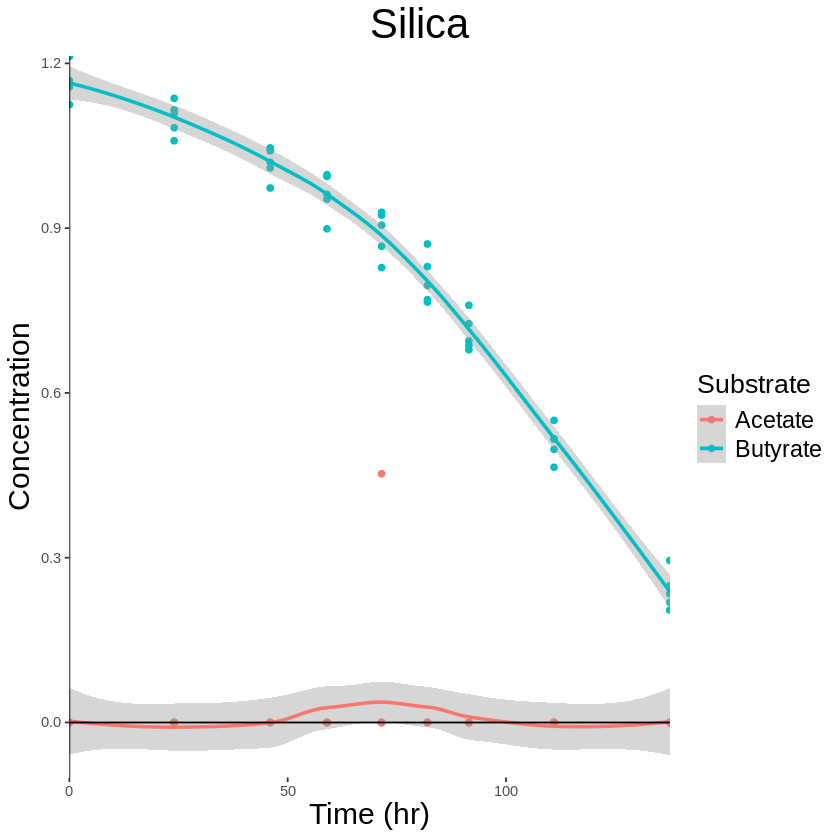

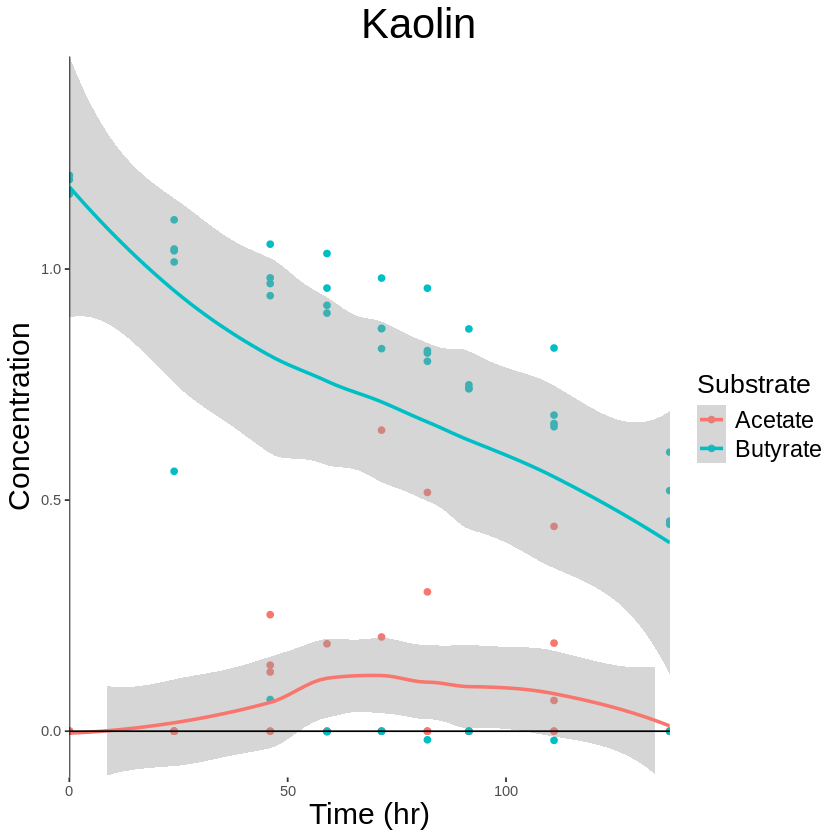

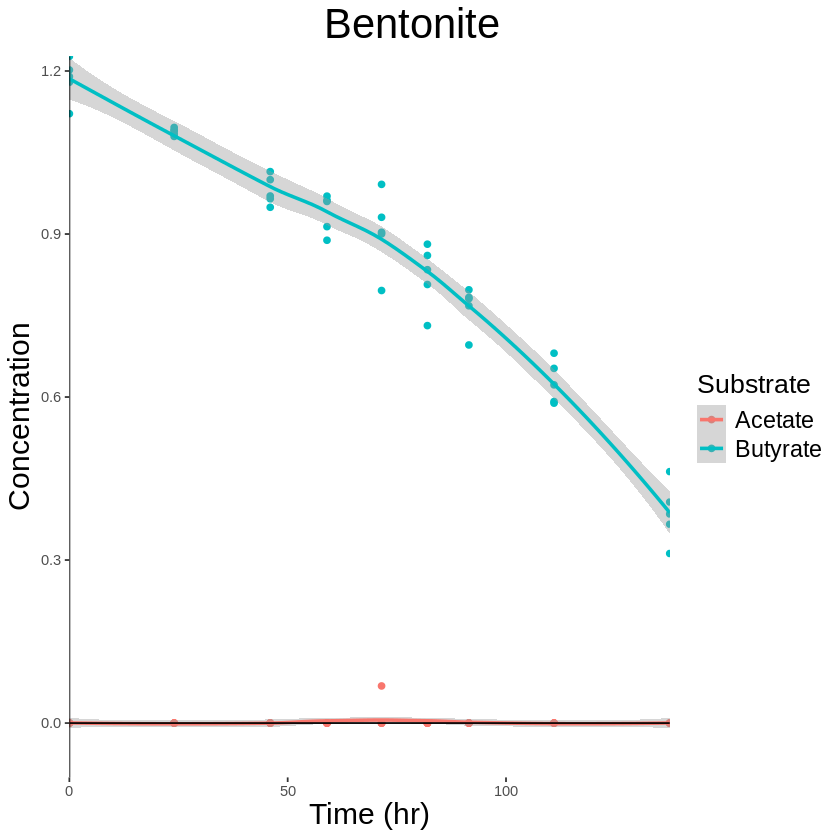
**Supplementary Figure S1**. Acetate production in R. palustris with and without clay supplementation with an initial substrate concentration of 1 g/L butyrate (n=5 replicates). In the suspended, silica, and bentonite conditions, negligible acetate is detected, suggesting that any acetate produced is immediately consumed. In the kaolin condition, some acetate production is observed; however, the absence of a clear trend similarly indicates a preference for acetate consumption.

| a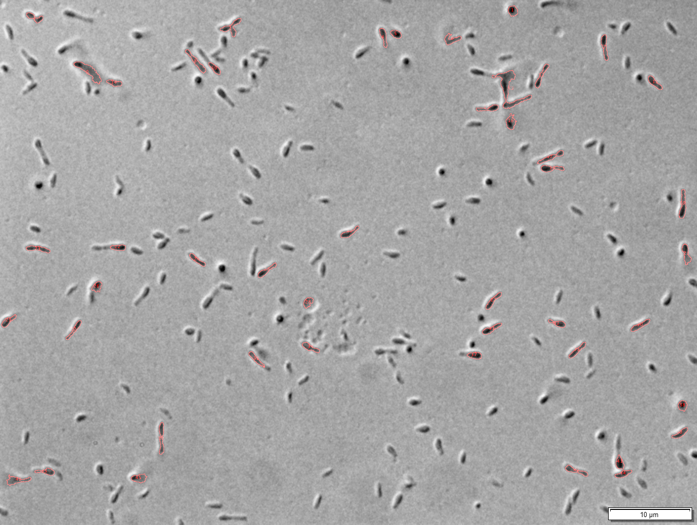 | b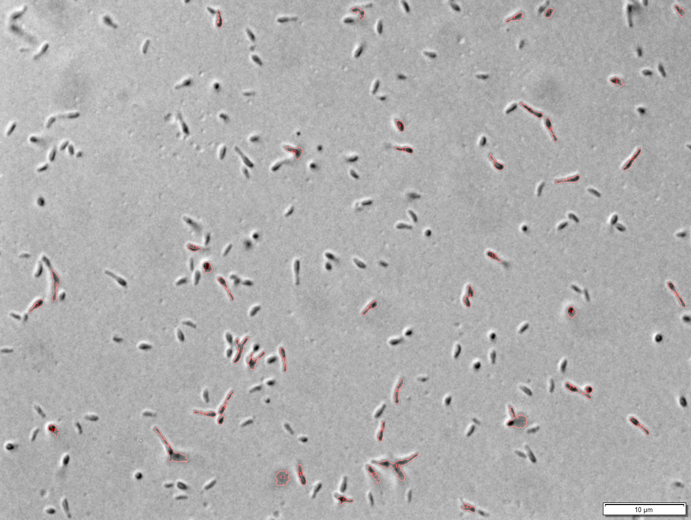 |
| --- | --- |
| c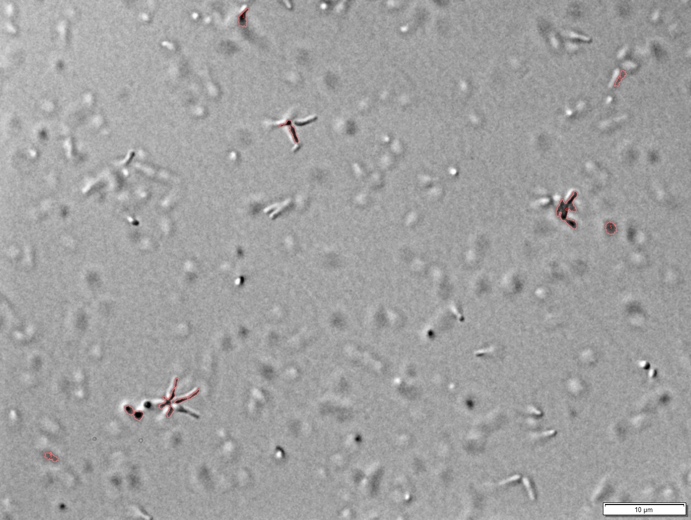 | d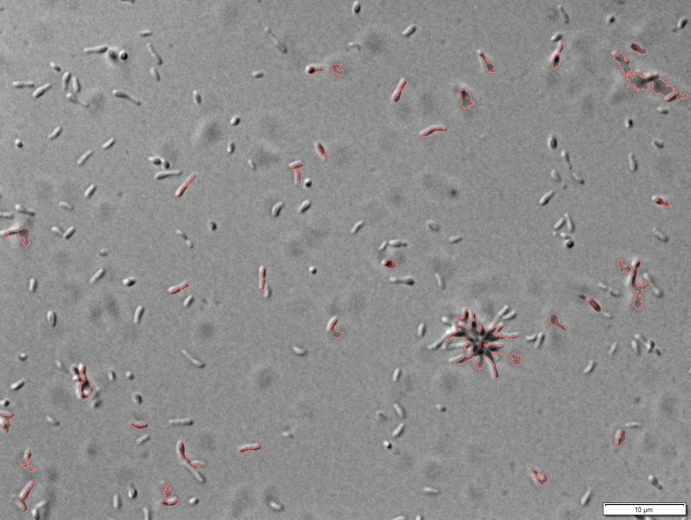 |
| e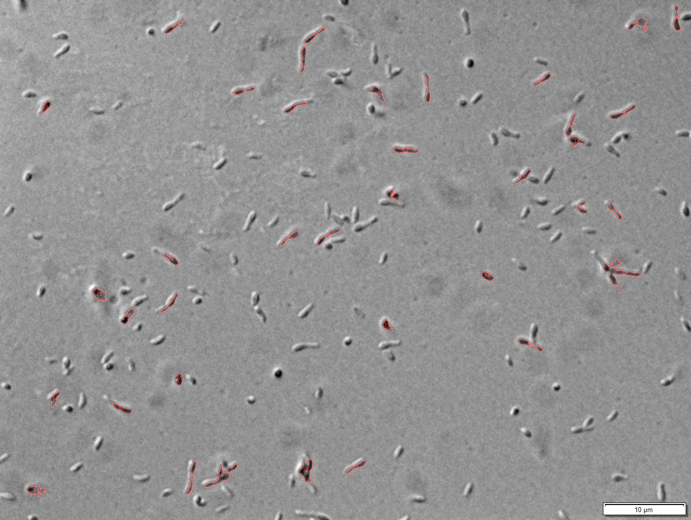 | f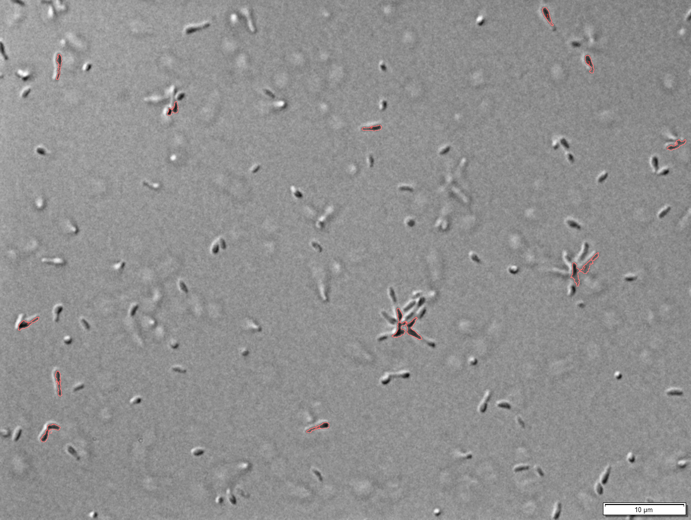 |

**Supplementary Figure S2**. Brightfield microscopy of R. palustris aggregates under suspended conditions at 100× magnification. Panels (a–f) show n=6 replicates. Images were converted to 8-bit, thresholded, and analyzed in Fiji. Scale bar = 10 µm. Aggregates were defined as two or more connected cells ≥0.1 µm².

| a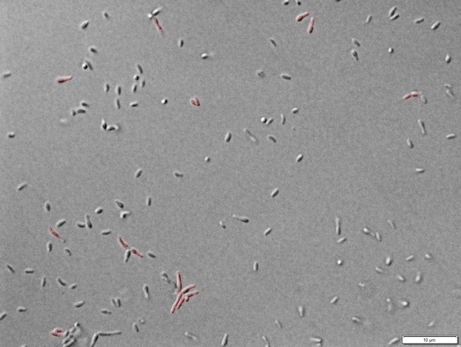 | b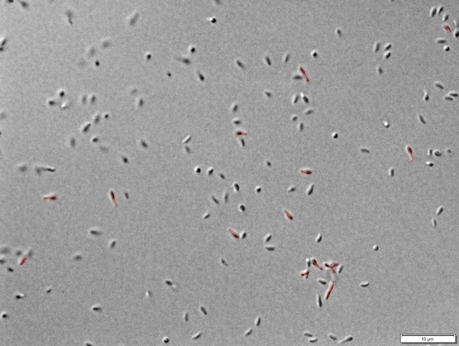 | c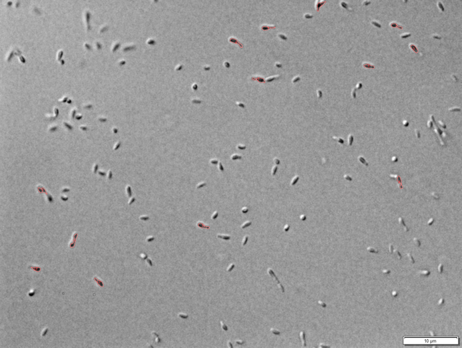 |
| --- | --- | --- |
| d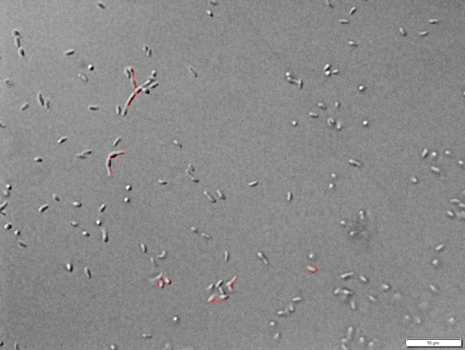 | e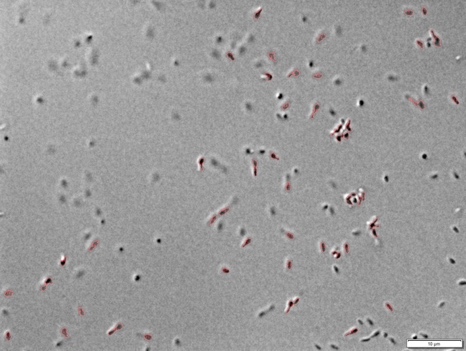 | f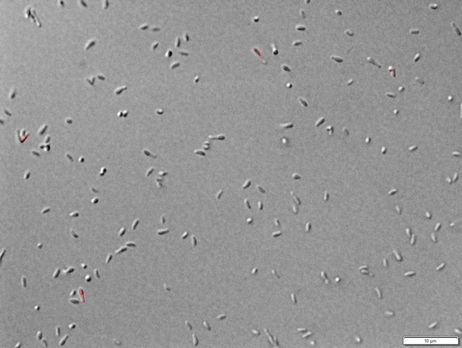 |
| g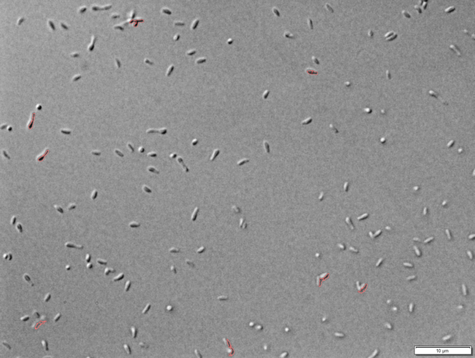 | h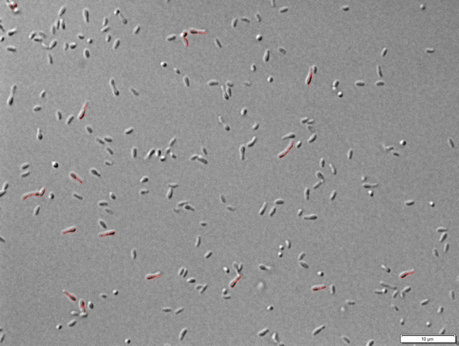 | i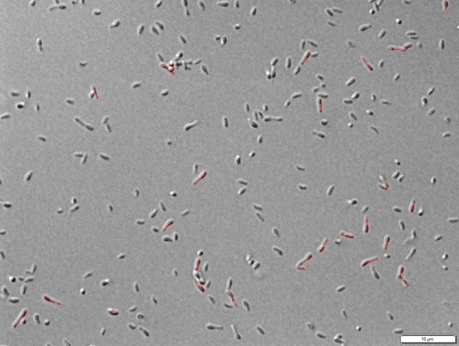 |

**Supplementary Figure S3**. Brightfield microscopy of R. palustris aggregates under silica conditions at 100× magnification. Panels (a–i) show n=9 replicates. Images were converted to 8-bit, thresholded, and analyzed in Fiji. Scale bar = 10 µm. Aggregates were defined as two or more connected cells ≥0.1 µm².

| a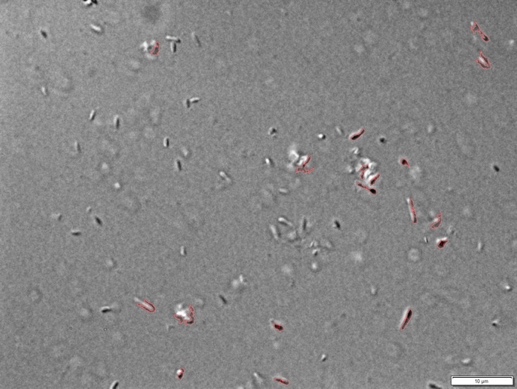 | b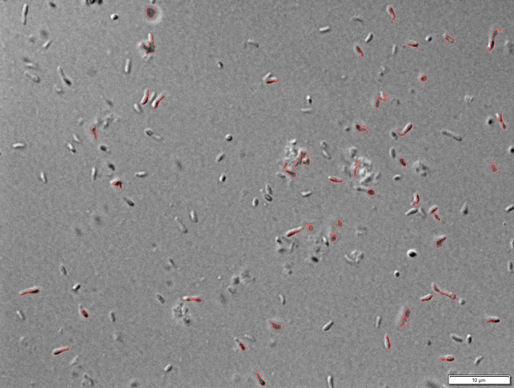 |
| --- | --- |
| c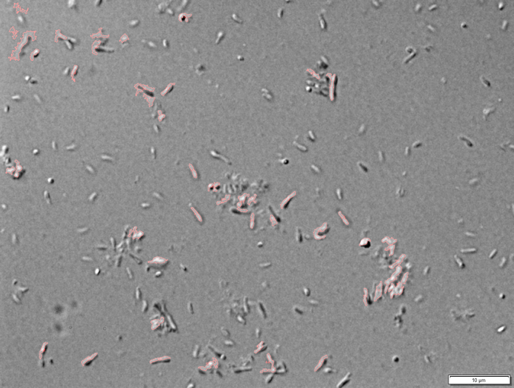 | d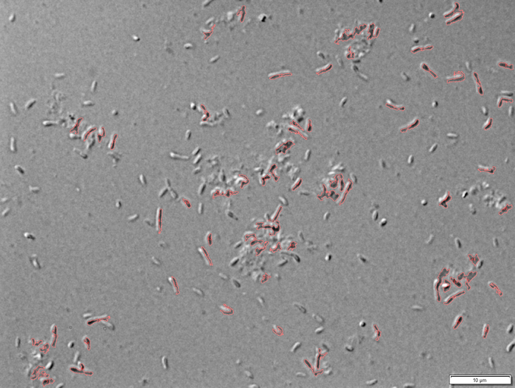 |
| e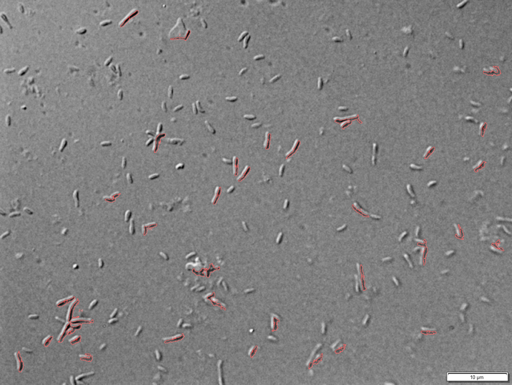 | f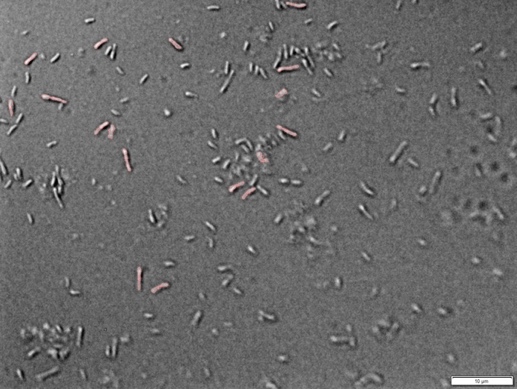 |
| g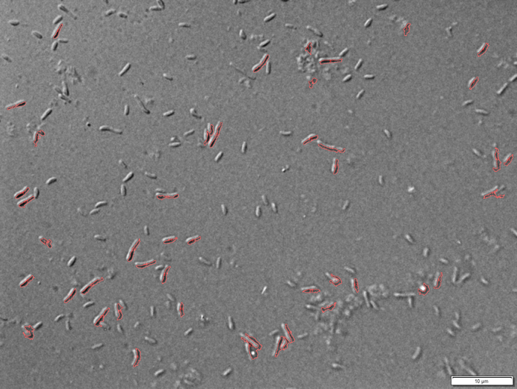 | h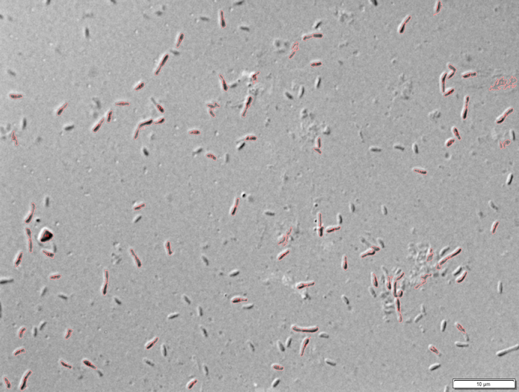 |

**Supplementary Figure S4**. Brightfield microscopy of R. palustris aggregates under bentonite conditions at 100× magnification. Panels (a–h) show n=8 replicates. Images were converted to 8-bit, thresholded, and analyzed in Fiji. Scale bar = 10 µm. Aggregates were defined as two or more connected cells ≥0.1 µm².

| a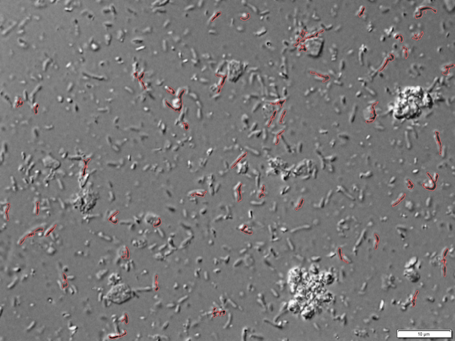 | b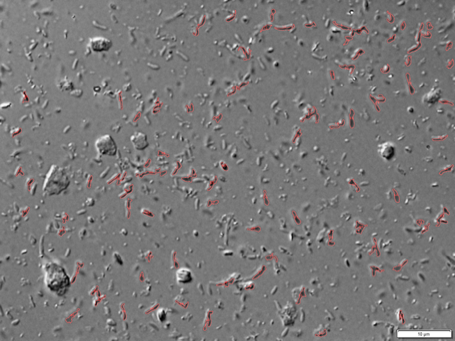 | c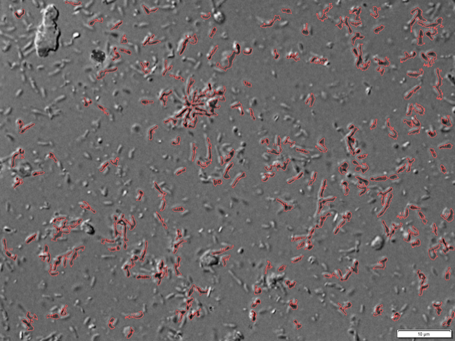 |
| --- | --- | --- |
| d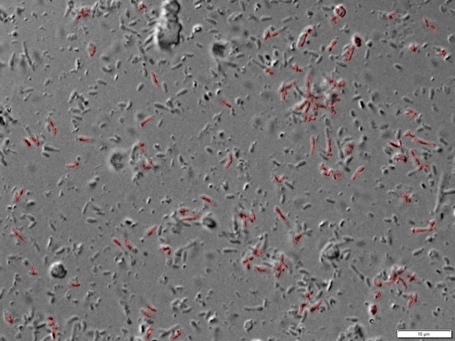 | e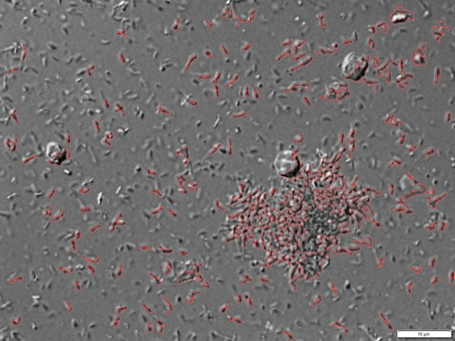 |  |
| f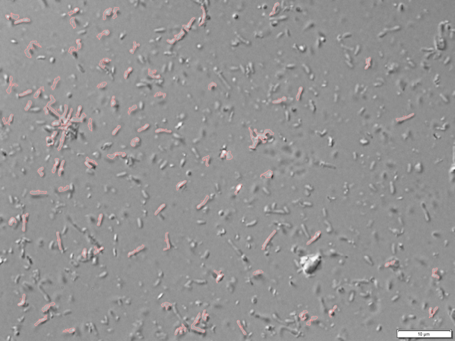 | g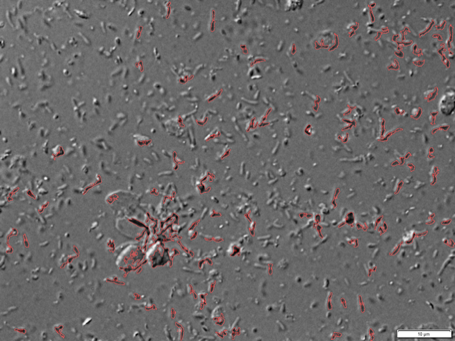 |  |

**Supplementary Figure S5**. Brightfield microscopy of R. palustris aggregates under kaolin conditions at 100× magnification. Panels (a–g) show n=7 replicates. Images were converted to 8-bit, thresholded, and analyzed in Fiji. Scale bar = 10 µm. Aggregates were defined as two or more connected cells ≥0.1 µm².


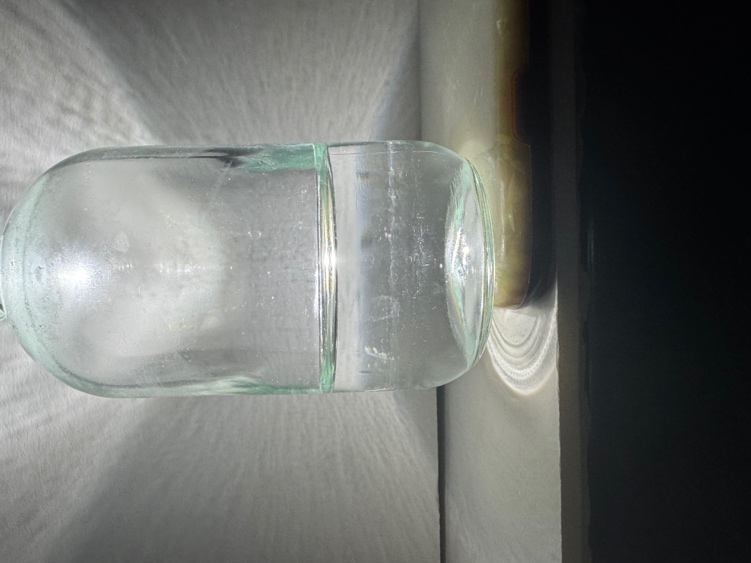

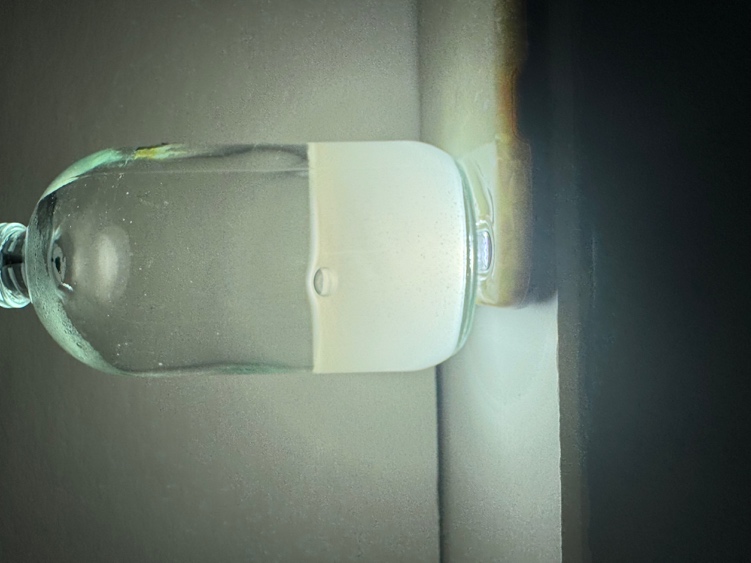

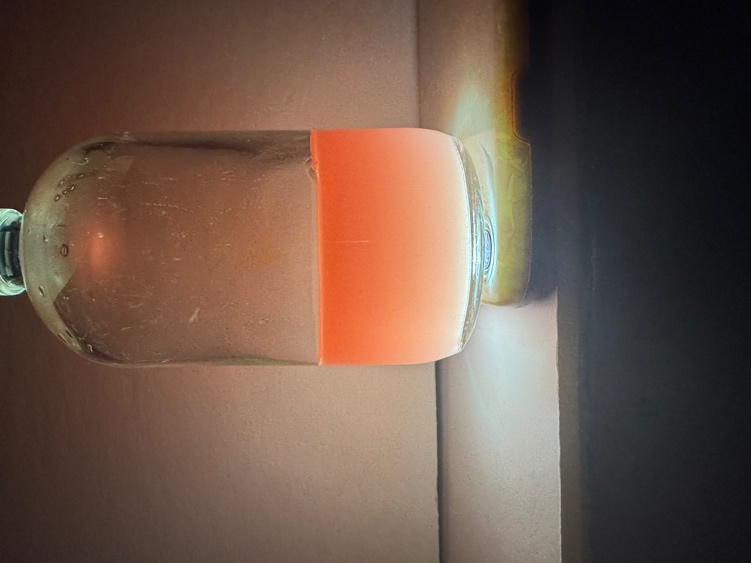

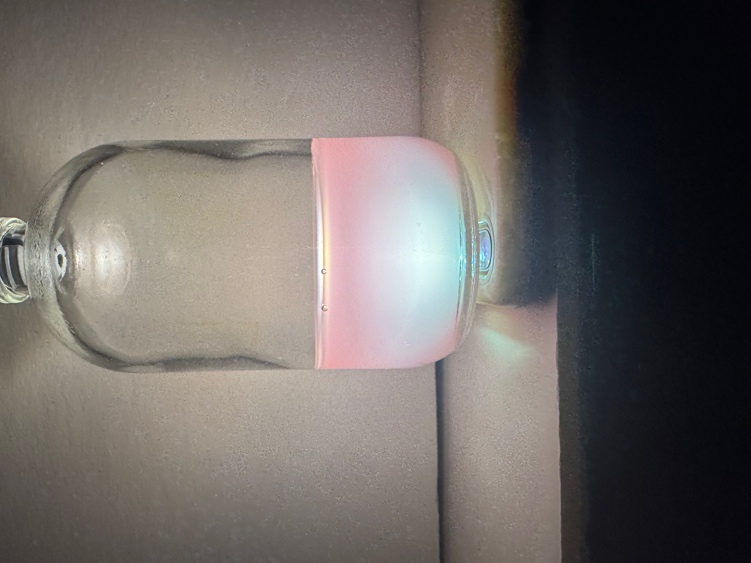


**Supplementary Figure S6.** Light distribution in serum bottles containing (from left to right): PM medium alone, PM medium with kaolin clay alone, PM medium with R. palustris cells (OD660 = 1.0), and PM medium with R. palustris cells (OD660 = 1.0) and kaolin clay. Bottles were irradiated from below, and measurements were taken from the side to assess lateral light transmission. The presence of R. palustris alone caused visible light attenuation due to cellular shading. In contrast, the addition of kaolin clay preserved lateral light transmission, even in the presence of cells, suggesting enhanced light scattering by kaolin particles.
