# Supplementary material for: Improving a photosynthetic bioprocess with a ubiquitous additive: Using clay powder in the cultivation of Rhodopseudomonas palustris
Source: Biotechnol Rep (Amst). 2025 Oct 8;48:e00930. doi: 10.1016/j.btre.2025.e00930 (PMC12550322; doi:10.1016/j.btre.2025.e00930)
Supplement: Supplementary file 2 [file mmc2.docx]

**Supplementary Tables**

**Supplementary Table S1.** High LI ANOVA and Games-Howell results for acetate concentrations across substrate conditions. Only comparisons to the suspended condition are shown. Mean differences represent the estimated difference in acetate concentration (g/L) between each pair. Bold entries indicate significant differences (p < 0.05).

| **Comparison** | **Mean Difference (g/L)** | **p-value** | **Significant?** |
| --- | --- | --- | --- |
| Bentonite vs Suspended | 3.90 | 0.354 | No |
| **Kaolin vs Suspended** | **20.2** | **0.000** | **Yes** |
| Silica vs Suspended | 1.83 | 0.836 | No |

**Supplementary Table S2.** High LI ANOVA and Games-Howell results for butyrate concentrations across substrate conditions. Only comparisons to the suspended condition are shown. Mean differences represent estimated differences in butyrate concentration (g/L). Bold entries indicate statistical significance (p < 0.05).

| **Comparison** | **Mean Difference (g/L)** | **p-value** | **Significant?** |
| --- | --- | --- | --- |
| Bentonite vs Suspended | 7.28 | 0.789 | No |
| Kaolin vs Suspended | 13.4 | 0.374 | No |
| Silica vs Suspended | 8.01 | 0.740 | No |

**Supplementary Table S3.** Low LI ANOVA and Games-Howell results for acetate concentrations across substrate conditions. Only comparisons to the suspended condition are shown. Mean differences represent the estimated difference in acetate concentration (g/L) between each pair. Bold entries indicate significant differences (p < 0.05).

| **Comparison** | **Mean Difference (g/L)** | **p-value** | **Significant?** |
| --- | --- | --- | --- |
| Bentonite vs Suspended | 16.4 | 0.274 | No |
| **Kaolin vs Suspended** | **25.3** | **0.039** | **Yes** |
| Silica vs Suspended | 8.51 | 0.881 | No |

**Supplementary Table S4.** Low LI ANOVA and Games-Howell results for butyrate concentrations across substrate conditions. Only comparisons to the suspended condition are shown. Mean differences represent estimated differences in butyrate concentration (g/L). Bold entries indicate statistical significance (p < 0.05).

| **Comparison** | **Mean Difference (g/L)** | **p-value** | **Significant?** |
| --- | --- | --- | --- |
| Bentonite vs Suspended | -15.4 | 0.564 | No |
| Kaolin vs Suspended | -11.7 | 0.433 | No |
| Silica vs Suspended | -16.8 | 0.453 | No |

**Supplementary Table S5.** Results of Mann–Whitney U tests comparing aggregate area distributions under Silica, Bentonite, and Kaolin conditions to the Suspended control. The U statistic and corresponding p-value are reported for each pairwise comparison. Statistically significant differences (p < 0.05) indicate that the distribution of aggregate areas under the given condition differs significantly from the Suspended control.

| **Comparison** | **U statistic** | **p-value** | **Significant?** |
| --- | --- | --- | --- |
| **Silica vs Suspended** | **15375.5** | **0.023992** | **Yes** |
| Bentonite vs Suspended | 29931.5 | 0.100792 | No |
| **Kaolin vs Suspended** | **62878.0** | **0.014501** | **Yes** |

***Supplementary Table S6.*** *Games-Howell post-hoc test following one-way ANOVA (F = 379.17,* p *< 0.0001) comparing PPFD values across four conditions. A significant reduction in PPFD was observed in the* Media + Bacteria *condition compared to all other groups, suggesting that the addition of kaolin mitigated the light attenuation effect caused by bacterial presence.*

| **Group 1** | **Group 2** | **Mean Diff** | **p-adj** | **95% CI Lower** | **95% CI Upper** | **Significant** |
| --- | --- | --- | --- | --- | --- | --- |
| **Media** | **Media + Bacteria** | **13.75** | **0.003** | **12.09** | **15.41** | **Yes** |
| Media | Media + Bacteria + Kaolin | 0.64 | 0.743 | -1.05 | 2.33 | No |
| Media | Media + Kaolin | –1.40 | 0.318 | -0.39 | 3.18 | No |
| **Media + Bacteria** | **Media + Bacteria + Kaolin** | **-13.11** | **<0.001** | **-13.66** | **-12.56** | **Yes** |
| **Media + Bacteria** | **Media + Kaolin** | **-12.35** | **<0.001** | **-13.16** | **-11.55** | **Yes** |
| Media + Bacteria + Kaolin | Media + Kaolin | 0.76 | 0.235 | -0.10 | 1.62 | No |
